# Ameliorative effects of Fingolimod (FTY720) on microglial activation and psychosis-related behavior in short term cuprizone exposed mice

**DOI:** 10.1186/s13041-023-01047-5

**Published:** 2023-07-12

**Authors:** Siyao Li, Koki Sakurai, Masahiro Ohgidani, Takahiro A. Kato, Takatoshi Hikida

**Affiliations:** 1 Laboratory for Advanced Brain Functions, Institute for Protein Research, Osaka University, Suita, Osaka Japan; 2 Graduate School of Frontier Biosciences, Osaka University, Suita, Osaka Japan; 3 Present Address: Laboratory of Protein Profiling and Functional Proteomics, Institute for Protein Research, Osaka University, Suita, Osaka, Japan; 4 Department of Functional Anatomy and Neuroscience, Asahikawa Medical University, Hokkaido, Japan; 5 Department of Neuropsychiatry, Graduate School of Medical Sciences, Kyushu University, Fukuoka, Japan

**Keywords:** Short-term cuprizone exposure, Psychosis, Fingolimod, Microglial activation, Neuroinflammation

## Abstract

Schizophrenia is a psychiatric disorder that affects around 1% of the population in widespread populations, with severe cases leading to long-term hospitalization and necessitation of lifelong treatment. Recent studies on schizophrenia have highlighted the involvement of inflammatory and immunoregulatory mechanisms with the onset of symptoms, and the usage of anti-inflammatory treatments are being tested against periods of rapid psychosis. In the central nervous system, microglia are the innate immune population which are activated in response to a wide range of physical and psychological stress factors and produce proinflammatory mediators such as cytokines. Microglial activation and neuroinflammation has been associated to numerous psychiatric disorders including schizophrenia, especially during psychotic episodes. Thus, novel treatments which dampen microglial activation may be of great relevance in the treatment of psychiatric disorders. Fingolimod (FTY720) is a drug used as an immunosuppressive treatment to multiple sclerosis. Recent clinical trials have focused on FTY720 as a treatment for the behavioral symptoms in schizophrenia. However, the mechanisms of Fingolimod in treating the symptoms of schizophrenia are not clear. In this study we use a recently developed neuroinflammatory psychosis model in mice: cuprizone short-term exposure, to investigate the effects of FTY720 administration. FTY720 administration was able to completely alleviate methamphetamine hypersensitivity caused by cuprizone exposure. Moreover, administration of FTY720 improved multiple measures of neuroinflammation (microglial activation, cytokine production, and leucocyte infiltration). In conclusion, our results highlight the future use of FTY720 as a direct anti-inflammatory treatment against microglial activation and psychosis.

**Supplementary Information:**

The online version contains supplementary material available at 10.1186/s13041-023-01047-5.

## Introduction

Schizophrenia is a psychiatric disorder that affects around 1% of the population in widespread populations, with severe cases leading to long-term hospitalization and necessitation of lifelong treatment [1–4]. Although the etiology of schizophrenia has not yet been fully elucidated yet, genetic and environmental evidence has pointed to dopaminergic disfunction and glutamatergic hypofunction as dominant hypotheses underlying symptoms [5]. Furthermore, recent studies have highlighted the involvement of inflammatory and immunoregulatory mechanisms in the onset of schizophrenia and related psychotic events [6, 7]. Based on hypotheses that psychotic episodes result from acute exacerbation of neuroinflammatory status, usage of anti-inflammatory treatments against periods of rapid psychosis are being investigated [8]. Such use of anti-inflammatory treatment has been supported by further evidence of anti-inflammatory drugs alleviating symptoms in patients with early-stage schizophrenia [9]. Despite such clinical evidence demonstrating the effectiveness of anti-inflammatory treatments, the causality and mechanism of neuroinflammatory events underlying psychosis-related behavioral deficits are yet to be fully uncovered.

Microglia are the primary innate immune population in the central nervous system (CNS), with known implications in brain development, neural network maintenance, and neural injury repair [10, 11]. Microglia are activated in response to a wide range of physical and psychological stress factors, which trigger the production of proinflammatory cytokines and reactive oxygen species [12], and lead to neuronal degeneration and/or white matter abnormality in the long term [13, 14]. In humans, microglial activation has been associated to various psychiatric and neurological disorders such as schizophrenia [13], Alzheimer's disease (AD) [15], Parkinson's disease (PD) [16], Amyotrophic lateral sclerosis (ALS) [17], and Multiple sclerosis (MS) [18]. As an investigative treatment to psychiatric disorders, minocycline, a widely used antibiotic, is known to inhibit microglial activation and have an ameliorative effect on the clinical performance in schizophrenic patients [19–21]. Thus, novel treatments which dampen microglial activation may be of great relevance in the treatment of psychiatric disorders [12, 14].

A particular treatment of recent interest regarding neuroinflammation is Fingolimod (FTY720). Known as an immunosuppressive treatment to MS, the active, phosphorylated form of Fingolimod depletes circulatory lymphocytes after administration by agonizing lymphocytic sphingosine 1-phosphate (S_1_P) receptors, resulting in reduced infiltration of autoreactive lymphocytes into the brain parenchyma [22–24]. Other than in lymphocytic populations, S_1_P receptors are expressed in most neuronal lineages and resident CNS cells, especially neural-derived glia and neurons [23, 24]. Moreover, Fingolimod has been reported to interact with other targets expressed in the CNS such as Transient Receptor Potential Cation Channel Subfamily M Member 7 (TRMP7), Histone Deacetylase, and Protein phosphatase 2A (PP2A), functioning as a potent inhibitor of microglial activation in culture [25]. In clinical trials, the efficacy of Fingolimod against patients with schizophrenia reemphasized the importance of treating dysregulated inflammatory states during acute psychotic exacerbations [8]. However, the mechanisms of Fingolimod in treating psychosis-related behavioral symptoms, especially in relation to neuroinflammation is not understood and calls for investigation using animal models to elucidate the pharmacological pathways targeted by FTY720.

Cuprizone short-term exposure is a recently developed neuroinflammatory psychosis model in mice which utilizes acute administration of cuprizone (Oxalic acid bis(cyclohexylidenehydrazide)), a copper depleting agent that induces oligodendrocytic damage through mitochondrial dysfunction [26, 27]. Short-term exposure to cuprizone has been shown to induce behavioral abnormalities including hypersensitivity to psychostimulants (methamphetamine and phencyclidine) and deficits in short-term memory related tasks [28], most likely associated with increased expression of proinflammatory cytokine interleukin 6 (IL-6) in the hippocampus [29]. In this study, we utilized this neuroinflammatory psychosis model mouse to investigate the effects of FTY720 administration on behavioral abnormalities, microglial activation status, and proinflammatory induction.

## Results

### Sample groups

To investigate the effects of FTY720 on short-term cuprizone treated mice, 7 week old C57BL/6NJcl mice were fed either control (Cont) or cuprizone (CUP) chow for 1 week and administered either Vehicle (Veh) or FTY720 (FTY) (4 Groups: Cont-Veh, Cont-FTY, CUP-Veh, CUP-FTY; Additional file 4: Fig. S1).

### FTY720 inhibited methamphetamine induced hyperactivity in short term cuprizone treated mice

The psychiatric symptoms of chronic methamphetamine addiction closely resemble the positive symptoms of schizophrenia, and schizophrenic patients are more prone to the effects of methamphetamine [30]. Similarly, short-term cuprizone exposure induces behavioral deficits in mice that are in line with human behavioral symptoms, including hypersensitivity to acute methamphetamine administration [29]. We investigated the effects of FTY720 on such increases in methamphetamine sensitivity in a methamphetamine-induced locomotion test.

**Fig. 1:**
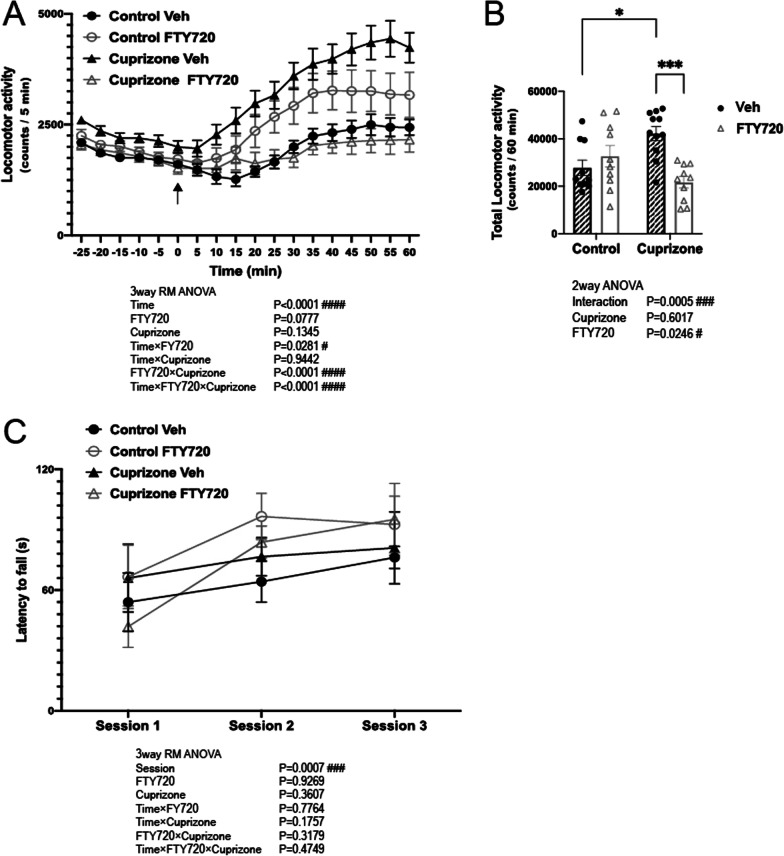
Effects of FTY720 on behavioral changes in short-term cuprizone-exposed mice. **A**, **B** Effects of FTY720 on locomotor activity in mice after methamphetamine administration. Mice were placed in a circular open field for 30 min to measure baseline locomotion, whereafter an intraperitoneal injection of methamphetamine (2 mg/kg) was administered to the mice and locomotion was measured for 60 min. **A** The locomotion (ambulatory counts) for 5-min bin within the total 90 min session (− 30 to 0 min before and 0 to 60 min after methamphetamine injection). Arrows indicate methamphetamine injection time. **B** Total locomotion (cumulative horizontal ambulatory counts) after injection of methamphetamine (0-60 min). n = 10–11 per group. **C** Rotarod measurement of basal locomotor function. The latency to fall from the rotarod (s) with maximum latency set at 120 s (n = 5 per group). All values are Mean ± SEM. Tukey test *p < 0.05, ***p < 0.001. **A**, **C** 3way RM ANOVA. **B** 2way ANOVA ^#^p < 0.05, ^###^p < 0.001, ^####^p < 0.0001

A significant interaction between Cuprizone exposure and FTY720 treatment was observed on locomotion after methamphetamine administration, with short-term cuprizone-exposed mice (CUP-Veh) showing significantly higher levels of locomotion compared to the control chow group (Cont-Veh) (Fig. 1A, B). Moreover, a significant effect of FTY720 on locomotion was observed, where the significant increase in locomotor activity in the CUP-Veh group was suppressed in the FTY720 treated Cuprizone group (CUP-FTY), to levels similar to the control chow group (Cont-Veh) (Fig. 1A, B). There was no significant effect of FTY administration between groups that were not exposed to cuprizone (Cont-Veh and Cont-FTY) (Fig. 1A, B). Examination of the basal motor functions of the 4 groups of mice in the rotarod test showed no significant effect of either Cuprizone or FTY on motor ability between groups (Fig. 1C). Evaluation of spatial memory using the Y-maze test showed that short-term cuprizone exposure does not affect total locomotion and arm entry. Although there was no significant difference in correct alternation amongst groups, a downward trend was seen in cuprizone exposed groups (Additional file 4: Fig. S2).

### FTY720 inhibits microglial activation in the hippocampus

**Fig. 2:**
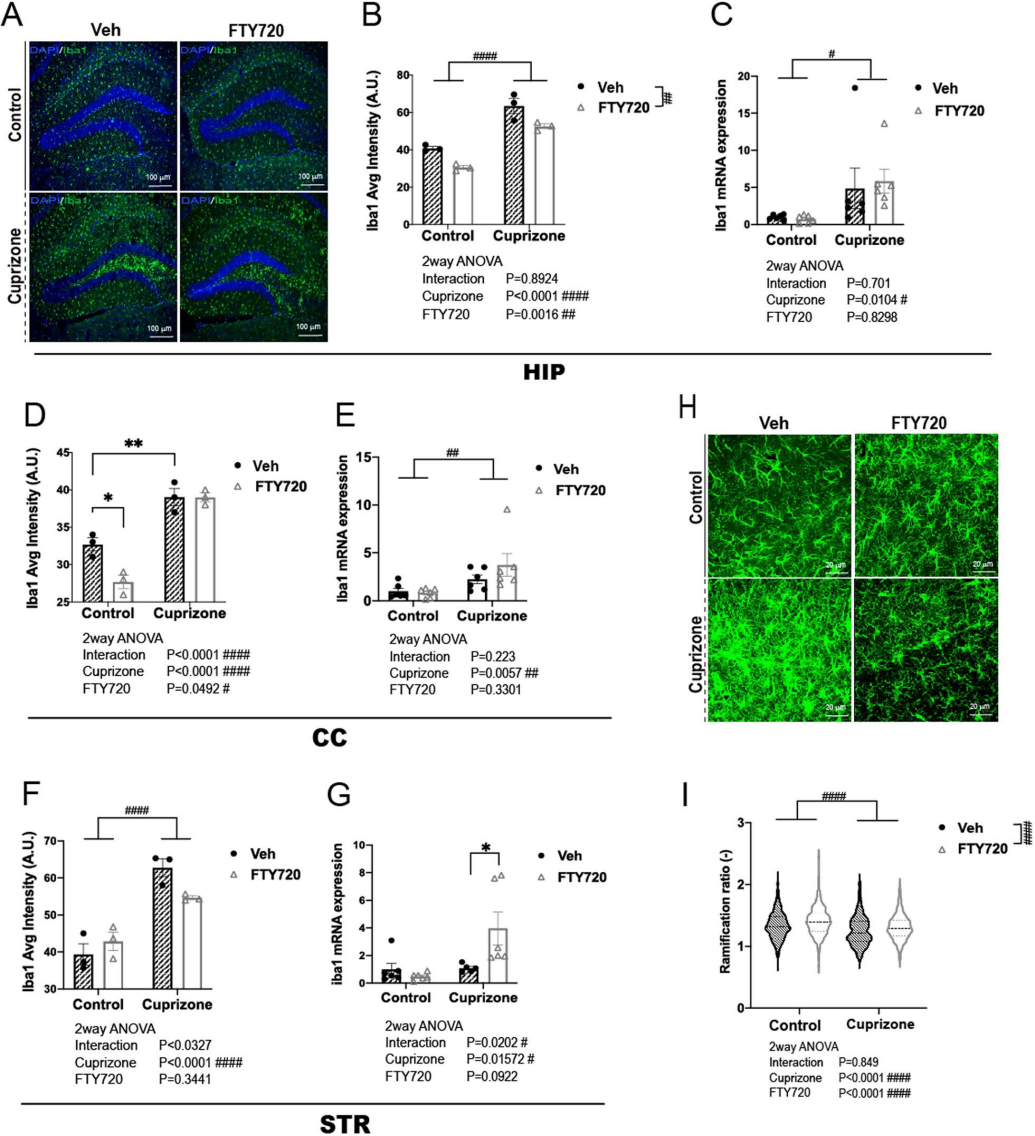
Effect of FTY720 on microglial activation in short-term cuprizone-exposed mice. **A**–**C** HIP. **A** Representative image of Iba1 immunostaining in the HIP. Blue: DAPI nuclear stain, Green: Alexa488 (Iba1). **B** Fluorescence intensity measurement of Iba1 signal in the HIP (n = 3 per group). **C** mRNA expression levels of Iba1 in the HIP. βActin was used for internal control, and samples were normalized to Cont-Veh. (n = 6 per group). **D**, **E** CC. **D** Fluorescence intensity measurement of Iba1 signal in the CC (n = 3 per group). **E** mRNA expression levels of Iba1 in the CC. **F**, **G** STR. **F** Fluorescence intensity measurement of Iba1 signal in the STR (n = 3 per group). **G** mRNA expression levels of Iba1 in the STR. βActin was used for internal control, and samples were normalized to Cont-Veh. (n = 6 per group). **H**, **I** Three-dimensional morphology analysis. **H** Representative image of z stack imaging. **I** Microglial ramification ratio. **B**–**G** Mean ± SEM. Tukey test *p<0.05, **p < 0.01. 2way ANOVA ^#^p < 0.05, ^##^p < 0.01, ^####^p < 0.0001. **I** Violin Plot Min-Max values. Dotted lines indicate first, second, and third quartiles. 2way ANOVA ^####^p < 0.0001

Glial activation induces the expression and release of cytokines, and postmortem brain studies of schizophrenic patients have reported hyperactivation of microglia [31, 32]. In addition, increased expression levels of the microglial gene ionized calcium binding adapter molecule 1 (Iba1), indicative of microglial activation, have been demonstrated in short-term cuprizone-exposed mice [29]. Therefore, we measured microglial activation by immunohistochemical staining for Iba1 protein (Fig. 2A, Additional file 4: Fig. S3) and assessed the effects of cuprizone and/or FTY720 treatment. In addition, we assessed for Iba1 mRNA gene expression in Hippocampus (HIP), Corpus Callosum (CC), and Striatum (STR) using RT-qPCR.

Significant effects of Cuprizone and FTY administration was observed on Iba1 signal levels in the HIP, CC, and STR, with Cuprizone administration causing marked increases of Iba1 signal, indicating increased microglial activation in the exposed groups (CUP-Veh and CUP-FTY) (Fig. 2B, D, F). In the HIP, FTY720 administration significantly decreased Iba1 levels in both Cont and CUP groups (Fig. 2B). In comparison, in the CC and STR, FTY720 was only effective in decreasing Iba1 levels in the Cont group (Fig. 2D, F). The expression levels of Iba-1 mRNA showed significant increases in HIP, CC, and STR as a result of cuprizone exposure but were not significantly affected by FTY720 administration (Fig. 2C, E, G).

To assess microglial activation in further detail, microglial morphology in the HIP was analyzed in three dimensions using high magnification, z-stacked microscopy (Fig. 2H, I). A significant effect of both Cuprizone administration and FTY720 was observed on microglial ramification ratios, a measure of microglial activation state (Fig. 2I). In the cuprizone exposed groups (CUP-Veh, CUP-FTY), there was a lower ramification ratio compared to groups with control chow (Cont-Veh, Cont-FTY), indicating ameboid-like morphological characteristics of activated microglia. FTY720 was effective in significantly increasing ramification ratio compared to Veh treated groups, indicating morphological characteristics of resting state microglia. Increased ramification ratio as a result of FTY720 administration was effective in restoring decreased ramification ratio in the CUP-Veh group to levels similar to untreated Cont-Veh group (Fig. 2I).

### FTY720 has anti-inflammatory effects in the hippocampus

Inflammatory activation in the brain induces the production and release of proinflammatory cytokines, mainly by glial cell populations, and cytokine alteration has been reported in schizophrenia patients [6, 7]. In addition, IL-6 mRNA levels are known to be increased in the HIP and STR of short-term cuprizone-exposed mice [29]. We examined the expression levels of proinflammatory cytokines interleukin 1 beta (IL-1β), IL-6, Tumor necrosis factor alpha (TNF-α), along with the expression level of anti-inflammatory cytokine interleukin 4 (IL-4) to evaluate changes in neuroinflammatory status induced by short-term cuprizone exposure and FTY720 treatment.

**Fig. 3:**
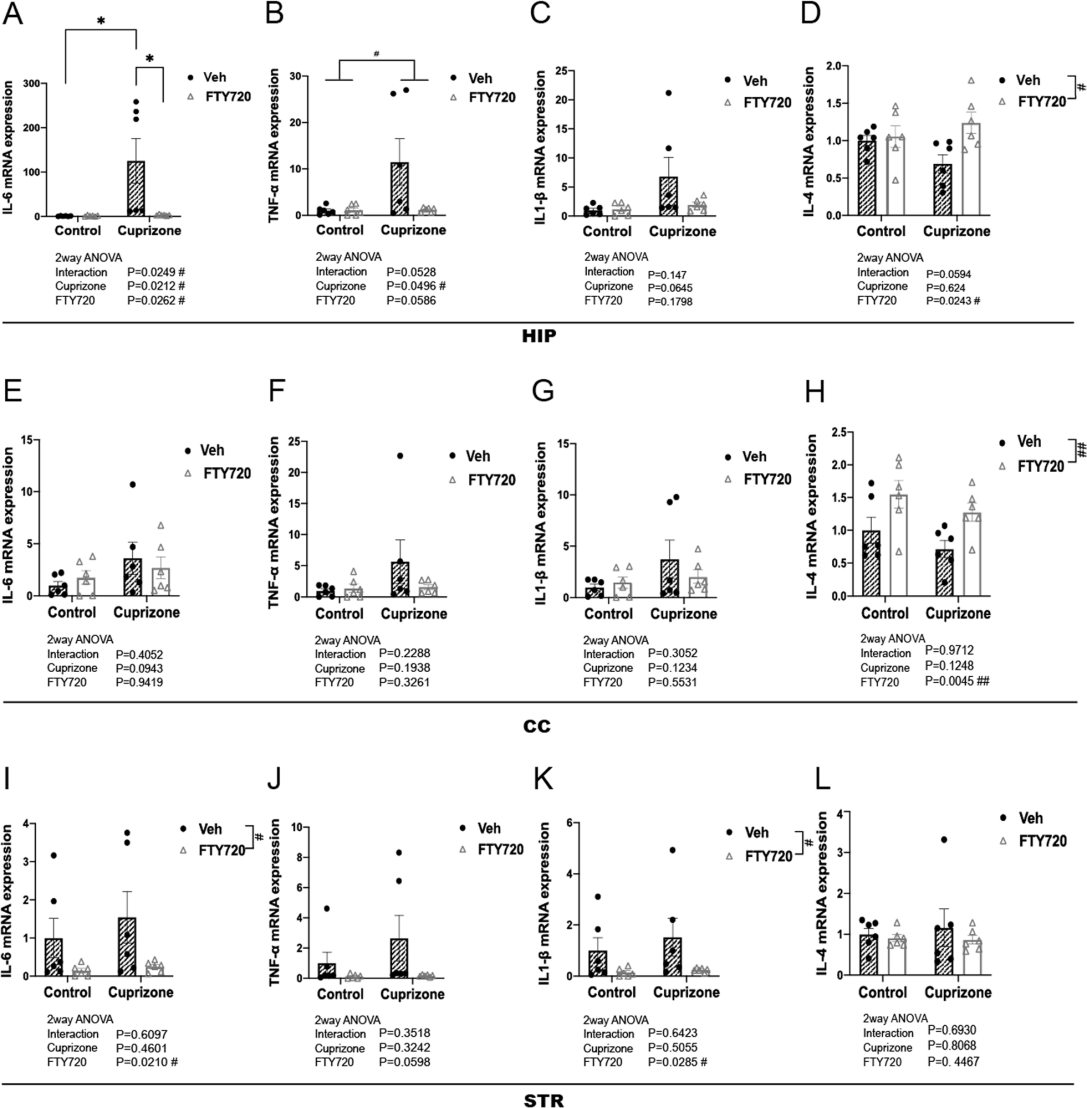
Effects of FTY720 on cytokine changes in short-term cuprizone-exposed mice. **A**–**D** mRNA expression levels of **A** IL-6, **B** TNF-a, **C** IL-1β, **D** IL-4 in the HIP. βActin was used for internal control, and samples were normalized to Cont-Veh. (n = 6 per group) **E**–**H** mRNA expression levels of **E** IL-6, **F** TNF-a, **G** IL-1β, **H** IL-4 in the CC. **I**–**L** mRNA expression levels of **I** IL-6, **J** TNF-a, **K** IL-1β, **L** IL-4 in the STR. βActin was used for internal control, and samples were normalized to Cont-Veh. (n = 6 per group). All values are Mean ± SEM. Tukey test *p < 0.05. 2way ANOVA ^#^p < 0.05, ^##^p < 0.01

In the HIP, a significant effect of Cuprizone treatment was seen in mRNA expression levels of proinflammatory cytokines IL-6 and TNF-α, with marked increases in the short-term cuprizone-exposed (CUP-Veh) group compared to other groups (Fig. 3A, C). Moreover, a significant effect of FTY720 along with a significant interaction between Cuprizone exposure and FTY720 administration was observed on IL-6 mRNA in the HIP, with FTY treatment significantly decreasing IL6 expression in the CUP-FTY group compared to the CUP-Veh group (Fig. 3A). In contrast, no significant effects of CUP and FTY was observed on expression levels of proinflammatory cytokines in the CC and STR (Fig. 3E–L). In both the HIP and CC, a significant effect of FTY720 administration was observed on the mRNA expression level of the anti-inflammatory cytokine IL-4, where FTY720 administration caused increased IL4 expression (Fig. 3D, H).

### FTY720 Short-term administration did not up-regulate leukocyte infiltration and myelin basic protein (MBP) expression

**Fig. 4:**
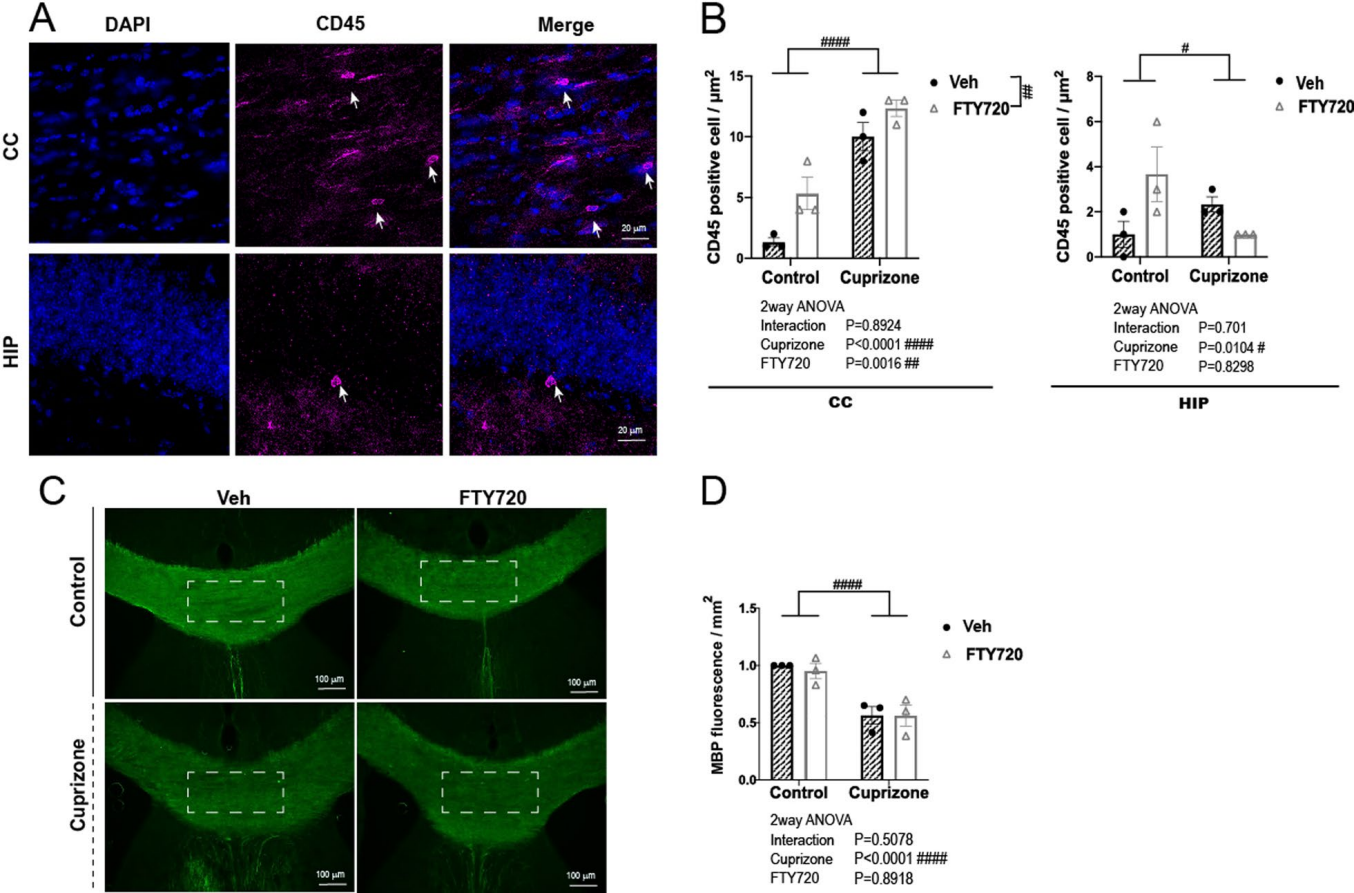
Effects of FTY720 on CD45^high^ cell population and MBP variability in short-term cuprizone-exposed mice. **A** Representative image of CD45 immunostaining in the HIP. Blue: DAPI nuclear stain, Green: Alexa488 (CD45). Arrows indicate CD45^high^ cells. **B** Number of CD45^high^ cells per area (µm^2^) in the CC and HIP. **C** Representative image of MBP immunostaining in the CC. Green: Alexa488 (MBP). **D** Fluorescence intensity of MBP immunostaining in the dotted area within the CC. (n = 3 per group). All values are Mean ± SEM. 2way ANOVA ^#^p < 0.05, ^##^p < 0.01, ^####^p < 0.0001

Neuroinflammation is known to cause disruption of the BBB, resulting in the infiltration of circulatory leukocytes into the brain. Such infiltration has been reported in patients with schizophrenia [33]. A significant increase in CD45^High^ cells [34] were observed in the CC of the short-term cuprizone-exposed mice, while no significant difference was observed in the HIP (Fig. 4A, B).

Furthermore, multiple studies have also reported oligodendrocyte and myelin abnormalities and associated gene dysregulation in the brains of patients with schizophrenia [35, 36]. FTY720 treatment in rodent demyelination models can dampen symptoms by enhancing myelin repair and upregulate expression levels of MBP, a major component of myelin [37, 38]. Therefore, we confirmed changes in MBP protein levels in the corpus callosum after short-term administration of FTY720 by immunohistochemistry. A significant effect of Cuprizone exposure was seen on MBP staining intensities with a marked decrease seen in cuprizone exposed groups, but no significant effect of FTY720 administration was observed (Fig. 4C, D).

## Discussion

In this study, we examined the effects of short-term treatment of FTY720 in an inflammatory mouse model of psychosis, demonstrating for the first time that FTY720, an immunosuppressant which functions as a potent inhibitor of microglial activation in culture [25], is effective in alleviating the schizophrenia-related behavioral symptoms and neuroinflammation in inflammatory psychosis model mice. In agreement with past studies, short-term cuprizone exposure induced methamphetamine hypersensitivity, microglial activation, increased proinflammatory cytokine production, leucocyte entry into the parenchyma, and decreased myelin protein expression in mice. We revealed that a short, 3 day treatment with FTY720 completely alleviates methamphetamine hypersensitivity and improves measures of neuroinflammation (microglial activation, cytokine production, and leucocyte infiltration). These results emphasize the usage of FTY720 as an anti-inflammatory treatment of psychosis and calls for further investigation of the neuroinflammatory and immunoregulatory mechanisms in the CNS related to psychosis and other psychiatric symptoms.

FTY720 was especially effective in reducing psychostimulant hypersensitivity, reducing levels of locomotion after methamphetamine treatment to levels similar to non-cuprizone-treated controls. Methamphetamine is a powerful and highly addictive stimulant with effects such as excitement, elimination of fatigue, mood enhancement, and euphoria, which functions by increasing synaptic dopamine concentration [38]. In human patients with schizophrenia, hypersensitivity to psychostimulants including methamphetamine have been reported [30, 39], suggesting overlap between methamphetamine targeted regions and ones responsible for the symptoms of schizophrenia. In this study, we chose minimal doses of methamphetamine (2 mg/kg) which do not induce marked increases in locomotion above basal levels in the untreated group (Cont-Veh). In contrast to controls and similar to human patients, short-term cuprizone-exposed mice were hypersensitive to methamphetamine treatment and exhibited marked increases in locomotor activity. In the short-term cuprizone-exposed mice treated with FTY720, hyperlocomotion was markedly decreased and reduced to similar levels as Cont-Veh.

Methamphetamine administration has been known to be closely associated to STR dopamine and glutamate levels [40]. Moreover, inflammatory conditions within the STR may alter synaptic connectivity and disrupt STR related circuitry [41]. Recent studies have provided evidence that methamphetamine administration is also relevant to HIP functions, where methamphetamine abuse has been reported to correlate with decreased HIP volume and white matter hypertrophy [42], as well as alterations of glial cell populations and expression of inflammatory factors in the HIP [43]. As shown in Figs. 2 and 3, our results are consistent with reports that short-term cuprizone exposure is associated with microglial activation and increased inflammatory cytokines in HIP and STR [29]. These findings suggest that short-term exposure to cuprizone may have caused some damage to the STR and HIP, resulting in behavioral abnormalities. Furthermore, suppressing HIP inflammation may improve behavioral abnormalities in response to methamphetamine.

Interestingly, in the Cont-FTY group, which was not exposed to cuprizone but treated with FTY720, an increasing trend in methamphetamine induced locomotor activity was observed. This suggests that at doses used in this study, FTY720 most likely does not suppress locomotor activity, as well as locomotor-function related dopaminergic signaling, and counteracts the cuprizone-induced increase in locomotion through other mechanisms such as altering glial functions related to synaptic maintenance [23, 44, 45].

Microglia are the innate immune cells of the CNS and are activated in response to various inflammatory/psychological stimuli such as tissue injury, infection, cytokine signaling and psychological stress. Microglial activation leads to upregulation of Iba1 as well as the release of response factors including proinflammatory mediators (ROS, cytokines, etc.) [12], and clinical studies have reported microglial hyperactivation in the brains of schizophrenic and suicidal patients [31, 32]. In line with such observations, increased signals of Iba1 protein were observed in the HIP, CC and STR of short-term cuprizone exposed mice, suggesting activated states of microglia. In particular, a strong increase in staining was observed in the HIP. On the other hand, the Cup-FTY group showed decreased Iba1 signal staining in the HIP, suggesting that FTY720 is effective in attenuating microglial activation in the HIP. A decrease in Iba1 signal as a result of FTY720 treatment was also observed in both the HIP and CC of the Cont-FTY group, which was not exposed to cuprizone, indicating that FTY720 can decrease basal levels of microglial activation in the absence of inflammatory stimulation. Interestingly, activation of microglia in the CC of cuprizone exposed mice could not be suppressed by FTY720. Cuprizone is known to affect different regions of the brain differentially, with the CC being a region especially targeted by cuprizone demyelination [46]. Similar to long-term exposure models of demyelination, regions surrounding the CC may be the primary region affected by acute cuprizone toxicity, thus having higher levels of microglial activation which make it difficult to attenuate with drug administration. Moreover, recent evidence suggests that there are multiple different subsets of microglia with differential gene expression profiles [47], and population specific accumulation in the CC may have decreased expression of FTY720 targets. It is possible that the different distribution of microglial subtypes specific to the hippocampus and corpus callosum may be related to the ameliorative effects against microglial activation. Further studies on the specific microglial subtypes and regions affected by cuprizone exposure may provide insight onto the populations targeted by FTY720.

In the present study, a marked increase in mRNA levels of IL-6 was observed in the HIP of the cuprizone exposed group. Moreover, trends towards increase of TNF-α and IL-1β was observed, though the changes were not statistically significant. In contrast, proinflammatory cytokines were not markedly increased in the CC and STR, with changes in all measured proinflammatory cytokines (IL-6, TNF-α, IL-1β) not being statistically significant. Such results may suggest region-specific differences in the surface receptor expression in residential cells [48] and consequent differences in susceptibility to neuroinflammation. Treatment with FTY720 significantly suppressed the elevation of IL-6 in the HIP. Changes in IL-6 as a result of cuprizone exposure and FTY720 administration coincided with observed changes in Iba1, suggesting that the anti-inflammatory effects of FTY720 are mediated through suppression of microglial activity. In our experiments, we saw significant increases of IL-4 expression as a result of FTY720 administration in both the HIP and CC. As IL-4 is a cytokine with potent anti-inflammatory effects [49, 50], the suppression of microglial activation and proinflammatory IL-6 expression may be due to increased IL-4 production, although the effects seem to differ between the HIP and CC.

Microglial activation and subsequent production of pro-inflammatory cytokines can disrupt the BBB [18], causing proinflammatory cell populations such as circulatory leukocytes to enter into the brain parenchyma [51]. Observation of cell populations with high expression of CD45 (CD45^High^) in the brain can visualize infiltrating leucocytic populations (mainly monocytes) [34]. In this study, a marked increase in CD45^High^ populations was observed in the CC of the cuprizone exposed groups, suggesting that short-term cuprizone exposure destabilizes the BBB, and allows for increased infiltration of circulatory leukocyte populations. Short-term treatment with FTY720 did not significantly alter the numbers of CD45^High^ populations in the HIP or CC. As an oral therapy for multiple sclerosis, the immunosuppressive effects of FTY720 restrict lymphocytes to lymphatic nodes, which is known to be mediated through the transient downregulation and degradation of the S_1_P receptor on circulatory lymphocytic populations [52]. It has been reported that FTY720 treatment lowers circulating leukocyte count and leukocyte recruitment in the CNS [53]. However, our results show that a short-term, three-day treatment does not significantly decrease brain parenchymal leucocytic (CD45^High^) populations. The neuroinflammatory state induced by short-term cuprizone exposure is significantly different from the reported LPS inflammation model, and it is possible that the effect of FTY720 administration is affected by differences in the degree of leukocyte infiltration [53]. Additionally, FTY720 has been shown to pass through the BBB and accumulate in the brain [23, 54] and can target microglial S_1_P receptors expressed in CNS resident cells. Moreover, there is evidence of FTY720 preventing BBB disruption by altering tight junction proteins that form the BBB [55]. Therefore, the anti-neuroinflammatory effects of FTY720 seen in this study can most likely reflects the direct effects of FTY720 on CNS resident cells or BBB forming cells.

Previous studies on schizophrenia have focused on myelin-related genes as a genetic risk factor of schizophrenia [37], and white matter abnormalities correlate with the stages of schizophrenia [56]. As FTY720 administration has been reported to have protective effects on models of inflammatory demyelination [38], we stained for MBP in the CC, a major white matter structure, to investigate if FTY720 administration can directly prevent oligodendrocytic damage. We observed a decrease in MBP staining in the cuprizone exposed groups, which FTY720 administration was not able to rescue. This suggests that the ameliorative effects of FTY720 are mainly anti-inflammatory and cannot directly rescue cuprizone-induced oligodendrocytic damage/loss, which is the primary cause of inflammation in this model.

In summary, this study utilized behavioral, immunohistochemical, and transcriptional analyses to evaluate the effects of short-term FTY720 administration on an inflammatory short-term cuprizone exposure model of psychosis. As a result, short-term administration of FTY720 improved disorder-related behavior and reduced inflammation by suppressing microglial activation in the HIP, highlighting its future use as a direct anti-inflammatory treatment against microglial activation and psychosis. These findings are consistent with the microglial hypothesis in psychosis such as schizophrenia and suggests that the suppression of microglial activation could alleviate psychosis pathology [13, 14]. It has been reported that neuroinflammation and/or the inflammasome pathway is associated with many neurological diseases [15–18, 57, 58]. New methods such as induced microglia-like (iMG) cells differentiated from human peripheral blood monocytes which exhibit microglia-like gene expression, branching morphogenesis, and phagocytic activity with various cytokine releases [59–61] have been developed to allow for evaluation in human tissue derived cells. iMG cells differentiated from schizophrenia patients may be used effectively to investigate the inhibitory effects of FTY720 on microglial activation in humans.

Short-term cuprizone exposure causes oligodendrocyte dysfunction, which includes disruption of mitochondrial and metabolic function, as well as ROS production [62]. Cuprizone exposure also causes amino acid depletion [62]. As a result, it has been reported that the synthesis of myelin-related proteins such as MBP may decrease [62]. Following oligodendrocyte injury, other glial cells including microglia are activated, and start to contribute to innate immune responses [62] by releasing proinflammatory cytokines. Such proinflammatory responses can cause neural inflammation, resulting in disease-related symptoms. It has been reported that increased microglial activation causes neuroinflammation and impairs hippocampal neurogenesis, neuronal synaptic morphology, and synaptic plasticity [63, 64]. Moreover, it has been reported that FTY720 attenuates microglial activity and downregulates the production of proinflammatory cytokines by activated microglia [65]. Such insights suggest the possibility of FTY720 suppressing neural cell dysfunction (inflammation, synaptic dysfunction, etc.) caused by short-term cuprizone exposure to alleviate pathological behavior. In addition to the microglial activity examined in this study, FTY720 is known to regulate immunoinflammatory responses through various mechanisms (Additional file 4: Fig. S4) [25, 66].

The full picture of human psychosis pathology still remains largely elusive and continues to be difficult to capture. Elucidation of the causes and development of therapeutic strategies likely requires further contributions from various fields including animal models, clinical studies, and human genetics. Short-term cuprizone exposure in mice, the model used in this study, can be an effective model of the neuroinflammatory symptoms of psychosis, and can be further utilized to study the connections between psychiatric symptoms and inflammatory phenomena due to their relevance in behavioral characteristics and state of neuroinflammation [32, 33]. Further information into the microglial populations altered by cuprizone and/or FTY720 and the cellular mechanisms of underlying the phenomena observed in this study may provide valuable insight on the connections between neuroinflammatory phenomena and behavioral alteration.

## Methods

### Animals

All animal experiments were performed in accordance with protocols of the Animal Experimentation Committee of the Institute for Protein Research, Osaka University. Male C57BL/6NJcl mice were obtained from CLEA Japan. Mice were maintained in a quiet environment with a controlled temperature of 24 ± 2 °C. During a 12-h light/dark cycle, mice had constant access to either a standard solid chow (AIN-93M, ORIENTAL YEAST CO., LTD.) or identical chow containing 2% w/w cuprizone (AIN-93M (99.8%) + cuprizone (0.2%), ORIENTAL YEAST CO.,LTD.) and water. After arriving at 6 weeks, mice were habituated to the standard chow for seven days, then subsequently divided into four groups (Cont-Veh, Cont-FTY, CUP-Veh, CUP-FTY) and fed either the standard diet (Cont-Veh, Cont-FTY) or the cuprizone-formulated diet (CUP-Veh, CUP-FTY) for seven days (Additional file 4: Fig. S1).

### Drug administration

FTY720 {2-amino-2-[2-(4-octylphenyl) ethyl]-1,3-propanediol} was obtained from Cayman Chemical. Powdered FTY720 was dissolved in dimethylsulfoxide-hydrochloride (DMSO-HCl) to make a 0.2 g/ml stock and stored at − 20 °C. At the time of use, the stock solution was diluted with physiological saline to produce a 0.2 mg/ml solution. Identical DMSO-HCl solution without FTY720 was dissolved in saline for control. Mice were treated for three days with daily intraperitoneal injections of FTY720 1 mg/kg starting four days after the start of cuprizone exposure (no injections on experimental day) (Additional file 4: Fig. S1).

### Behavioral analysis

#### Rotarod

An accelerating rotarod (ROTA ROD; UGO BASILE) was used, with the initial speed set at 4 rpm, acceleration at 20 rpm/min, and maximum speed at 40 rpm. The mouse was placed on the rotating rod in the direction opposite to the direction of rotation. The test was started after the mouse was able to stand and move upright on the rotarod. The duration of time till falling to the platform below was recorded, with the maximum test duration set to 120 s after the start of acceleration. Mice were subject to three trials in one day, performed 60 min apart.

#### Methamphetamine-induced locomotion test

Methamphetamine (Sumitomo Pharma) was dissolved in physiological saline and administered intraperitoneally at a final concentration of 2 mg/kg. Mice were left in the experimental room for over an hour before being transferred to a circular open field (diameter 40 cm, height 27 cm, grey plexiglass). After 30 min of baseline locomotor activity measurement, mice were given intraperitoneal injections of methamphetamine and locomotion were measured for 60 min after injection. Mouse movement was tracked, and distance traveled (cm/5 min) was measured using EthoVision XT (Noldus).

#### Y-maze test

The Y-maze test was administered according to Sakurai et al. [67], with minor modifications. In brief, the Y-maze consisted of 3 arms (42 cm long, 16 cm high and 12 cm wide) configured 120 degrees apart. Mice were placed in the center of the arm and allowed to move freely in the maze for 10 min. Mouse movements and arm entries were tracked, recorded, and counted using EthoVision XT (Noldus), and calculation of successful alternation rate was calculated using an R script (Additional file 1). Successful alternation was defined as entering all three arms consecutively.

### Immunohistochemistry

Immunohistochemistry was performed according to Aomine et al. [68], with minor modifications. Animals were deeply anesthetized with isoflurane prior to experimentation and perfused transcardially with phosphate-buffered saline (PBS, pH 7.4) followed by 4% Paraformaldehyde/PBS, and post fixed overnight in 4% Paraformaldehyde/PBS at 4 ℃. For cryoprotection, brains were placed in 15% sucrose/PBS for one day, after being moved to 30% sucrose/PBS for ~ 36 h at 4 ℃. Brains were frozen and embedded in embedding agent (TissueTek O.C.T compound, Sakura Finetek Japan Co., Ltd., Tokyo, Japan) and was sectioned at 40 μm using a cryostat microtome (Leica CM1860, Leica Biosystems). To avoid deformation, the sections were free-float-processed with extreme caution.

Sections were washed three times with PBS-T (0.3% Triton-X) and incubated overnight (4 ℃) in an antibody solution (0.3% Triton X PBS W/ 10% Normal Goat Serum (Thermo Fisher) + primary antibody). Primary antibodies used were Anti Iba1 (1:1000; Rabbit, FUJIFILM Wako, 019-19741), Anti CD45 (1:100; Mouse, BIO-RAD, MCA87) and Anti Myelin Basic Protein (1:500; Rabbit, Abcam, ab133620). After primary antibody staining, sections were washed three times with PBS and incubated in a secondary antibody solution (0.3% Triton X PBS W/0.5% Normal Goat Serum + secondary antibody) for 2 h (RT). Secondary antibodies used were Anti Rabbit IgG, Alexa Fluor™ 488 (1:500; goat, ThermoFisher, A-11034), Anti Mouse IgG, Alexa Fluor™ Plus 647 (1:100; Goat, ThermoFisher, A-32728). The sections were washed three times with PBS, mounted on a glass slide with Mounting Medium w/DAPI (Abcam, ab104139), coverslipped, and imaged with a Keyence BZ X810 microscope.

### Fluorescence intensity analysis

#### HALO analysis

Fluorescence intensity was analyzed using HALO™ (Indicalab). Three individual images at 40× magnification were obtained per region per one mouse were. Care was taken to select the same regions for each individual. Thresholds were set to automatically detect DAPI positive nuclei and Iba-1 positive signal and were kept the same for all measurements. Iba-1 fluorescence intensity was measured for all Iba-1 positive nuclei and was average Iba-1 fluorescence intensity was calculated per image analyzed by HALO. The average values of three measurements per individual was used for further analysis.

#### ImageJ analysis

Sections were stained by the method described above, then observed and photographed with a Keyence BZ X810 microscope. The captured images were imported into Image J, and the fluorescence intensity per area (300 µm*120 µm; indicated by dotted line) of the corpus callosum region of 3 sections for each individual was measured. Prior to statistical analysis, mean values were calculated for each group (n = 3 mice each) based on the measured values, and relative values were calculated using the mean value of control mice as 100%. Fluorescence intensity statistics results are shown as mean ± SEM.

#### 3D cell morphology analysis of microglia

Animals were deeply anesthetized with isoflurane prior to experimentation and were perfused transcardially with phosphate-buffered saline (PBS, pH 7.4), followed by a fixative (a mixture of 4.0% paraformaldehyde and 0.05% glutaraldehyde in 0.1 M PBS). The brains were left in situ for 2 h at room temperature and were then removed from the skull. After cryoprotection with 30% sucrose, brain blocks were cut into 40-μm-thick sections on a cryostat (Leica CM1950, Wetzlar, Germany). To avoid deformation, the sections were free-float-processed with extreme caution. Sections were blocked for 30 min with 1% bovine serum albumin (BSA) in PBS containing 0.3% Triton X-100 and 0.1% sodium azide at 4 °C. After blocking, the sections were incubated with Anti Iba1 (1:20000; Rabbit, FUJIFILM Wako, 019-19741) for 2 days at 4 °C. After washing with PBS, the sections were incubated with a mixture of Alexa488-conjugated donkey anti-rabbit IgG antibody (1: 300; Jackson ImmunoResearch Laboratories, West Grove, PA, USA) overnight at 4 °C. The sections were counterstained with DAPI and mounted with Vectashield (Vector Laboratories, Burlingame, CA).

Fluorescent images were acquired with a confocal laser scanning microscope (FV-1000-D; Olympus, Japan). Sections were randomly sampled and processed for immunohistochemical staining. The 3D hippocampal microglia images were created with a × 60 objective on an FV-1000-D at 0.5-μm intervals along the z-axis. Following acquisition, images were compiled and transformed into 3D microglial renderings using Imaris software (Bitplane, Zurich, Switzerland). The morphological microglial parameters (cell surface area and cell volume) were measured using the Imaris MeasurementPro function. The microglia ramification ratio was calculated as the ratio of cell surface area to cell volume [69]. The morphological parameters from 545 to 631 microglia in each group were calculated from ten sections per mouse (n = 3 mice each).

### Quantitative real-time PCR

Eight-week-old mice were deeply anesthetized with isoflurane, decapitated, and the entire brain was removed rapidly from the skull. The whole brain was sliced into 1 mm sections using a brain matrix (Brainscience Idea, Osaka, Japan), and the striatum, hippocampus, and corpus callosum were removed from sections using a razor blade. Removed sections were placed in RNAlater, kept at 4 ℃ overnight, and then frozen at − 80 ℃.

RNA was extracted from tissue using RNeasyMiniKit (Qiagen) and reverse transcribed into cDNA with the ReverTra Ace® qPCR RT Master Mix (Toyobo). Quantitative real-time PCR was performed using GeneAce SYBR® qPCR Mix a Low Rox (NIPPON GENE) reagent and QuantStudio 6 pro (Thermo Fisher). Quantification was done using a relative standard curve, and βActin was used as an internal control. qPCR run method and Primer information is shown in Additional file 2.

Prior to statistical analysis of quantitative real-time PCR data, the cuprizone group mRNA expression levels were normalized to the control group for each brain region. Fluorescence intensity and quantitative real-time PCR statistical results are shown as mean ± SEM.

### Statistical analysis

All statistical analysis was performed using Prism8 (GraphPad Software), except for Fig. 2G which was analyzed using the “rstatix” library in R. The number of individual samples used for each set of experiments is indicated in the results section and figure legends. Details of ANOVA and post-hoc analyses are provided in Additional file 3.

## Supplementary Information

**Additional file 1: ** Y-maze R script. R script to calculate succesful alternation rate for Y-maze.

**Additional file 2:** RT-qPCR Details. qPCR run method and primer information.

**Additional file 3:** Statistics Details. F and P values for ANOVAs, and P values for post-hoc tests.

**Additional file 4: Fig S1. **Experimental Timeline for Short-Term Cuprizone Exposure. 6 weeks old mice were habituated to the control diet (w/o cuprizone) for 7 days (− 7 to 0 days) before administration of the cuprizone-containing diet. FTY720 was administered intraperitoneally 4, 5 and 6 days after administration of the cuprizone containing diet. All mice were sacrificed on day 7. All experiments were performed in four groups: Cont-Veh, Cont-FTY720, CUP-Veh, CUP-FTY. **Fig S2. **Effects of FTY720 on short-term spatial memory in cuprizone-exposed mice. Y-maze test for short-term spatial memory was used to assess the effects of cuprizone exposure and the effects of FTY720 administration. Total distance. (B) Total arm entries. (C) Successful alternation rate. **Fig S3. **Representative image of immunostaining in hippocampus and corpus callosum. (A) Representative image of Iba1 immunostaining in the HIP. (B) Representative image of Iba1 immunostaining in the CC. Blue: DAPI nuclear stain, Green: Alexa488 (Iba1). An enlarged view of the top row is shown in the bottom row. **Fig S4.** Hypothesis of FTY720 mechanism of action in psychosis model mice. Short-term cuprizone exposure does not cause prominent demyelination, but specifically causes damage to oligodendrocytes. Such damage results in activation of glial populations including microglia, triggering the release of proinflammatory cytokines. Extracellular release of proinflammatory cytokines induces neuronal dysfunction and contributes to behavioral disorders such as psychosis. FTY720 administration inhibits microglial activation and regulates pro-inflammatory cytokine release, reduces neuronal damage, and improves psychosis-like behavior.

## Data Availability

All data needed to evaluate the conclusions in the paper are present in the paper. The datasets used and/or analyzed during the current study are available from the corresponding author on reasonable request.

## Abbreviations

IL-6: Interleukin 6
TNF-α: Tumor necrosis factor-α
CNS: Central nervous system
AD: Alzheimer's disease
PD: Parkinson's disease
ALS: Amyotrophic lateral sclerosis
MS: Multiple sclerosis
PET: Positron emission tomography
FTY720: Fingolimod
S_1_P: Sphingosine 1-phosphate
TRMP7: Transient receptor potential cation channel subfamily M member 7
PP2A: Protein phosphatase 2A
HIP: Hippocampus
CC: Corpus callosum
Iba1: Ionized calcium binding adapter molecule 1
IL1-β: Interleukin-1beta
IL-4: Interleukin 4
MBP: Myelin basic protein
BBB: Blood–brain barrier
STR: Striatum
