# Supplementary material for: Ameliorative effects of Fingolimod (FTY720) on microglial activation and psychosis-related behavior in short term cuprizone exposed mice
Source: Mol Brain. 2023 Jul 12;16:59. doi: 10.1186/s13041-023-01047-5 (PMC10339554; doi:10.1186/s13041-023-01047-5)
Supplement: Supplementary file 4 — Additional file 4: Fig S1. Experimental Timeline for Short-Term Cuprizone Exposure. 6 weeks old mice were habituated to the control diet (w/o cuprizone) for 7 days (− 7 to 0 days) before administration of the cuprizone-containing diet. FTY720 was administered intraperitoneally 4, 5 and 6 days after administration of the cuprizone containing diet. All mice were sacrificed on day 7. All experiments were performed in four groups: Cont-Veh, Cont-FTY720, CUP-Veh, CUP-FTY. Fig S2. Effects of FTY720 on short-term spatial memory in cuprizone-exposed mice. Y-maze test for short-term spatial memory was used to assess the effects of cuprizone exposure and the effects of FTY720 administration. Total distance. (B) Total arm entries. (C) Successful alternation rate. Fig S3. Representative image of immunostaining in hippocampus and corpus callosum. (A) Representative image of Iba1 immunostaining in the HIP. (B) Representative image of Iba1 immunostaining in the CC. Blue: DAPI nuclear stain, Green: Alexa488 (Iba1). An enlarged view of the top row is shown in the bottom row. Fig S4. Hypothesis of FTY720 mechanism of action in psychosis model mice. Short-term cuprizone exposure does not cause prominent demyelination, but specifically causes damage to oligodendrocytes. Such damage results in activation of glial populations including microglia, triggering the release of proinflammatory cytokines. Extracellular release of proinflammatory cytokines induces neuronal dysfunction and contributes to behavioral disorders such as psychosis. FTY720 administration inhibits microglial activation and regulates pro-inflammatory cytokine release, reduces neuronal damage, and improves psychosis-like behavior. [file 13041_2023_1047_MOESM4_ESM.docx]

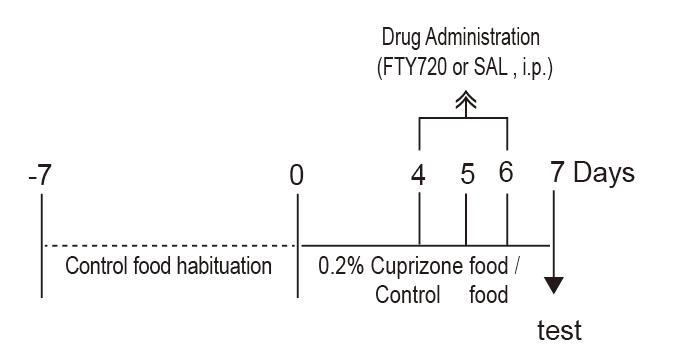


**Fig S1. Experimental Timeline for Short-Term Cuprizone Exposure**

6 week old mice were habituated to the control diet (w/o cuprizone) for 7 days (-7 to 0 days) before administration of the cuprizone-containing diet. FTY720 was administered intraperitoneally 4, 5 and 6 days after administration of the cuprizone containing diet. All mice were sacrificed on day 7. All experiments were performed in four groups: Cont-Veh, Cont-FTY720, CUP-Veh, CUP-FTY.

**
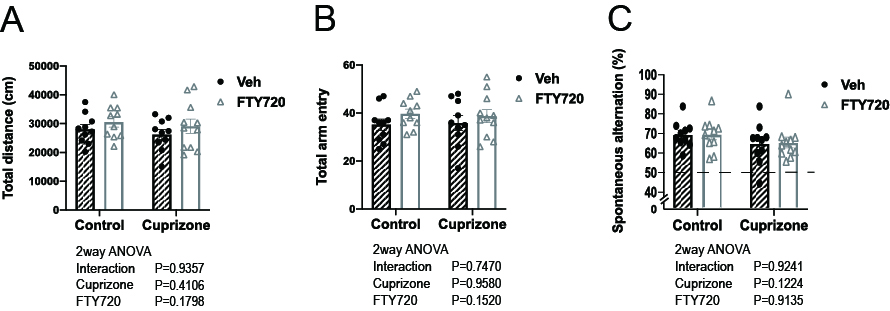
**

**Fig S2. Effects of FTY720 on short-term spatial memory in cuprizone-exposed mice.**

Y-maze test for short-term spatial memory was used to assess the effects of cuprizone exposure and the effects of FTY720 administration.

1. Total distance. (B) Total arm entries. (C) Successful alternation rate.


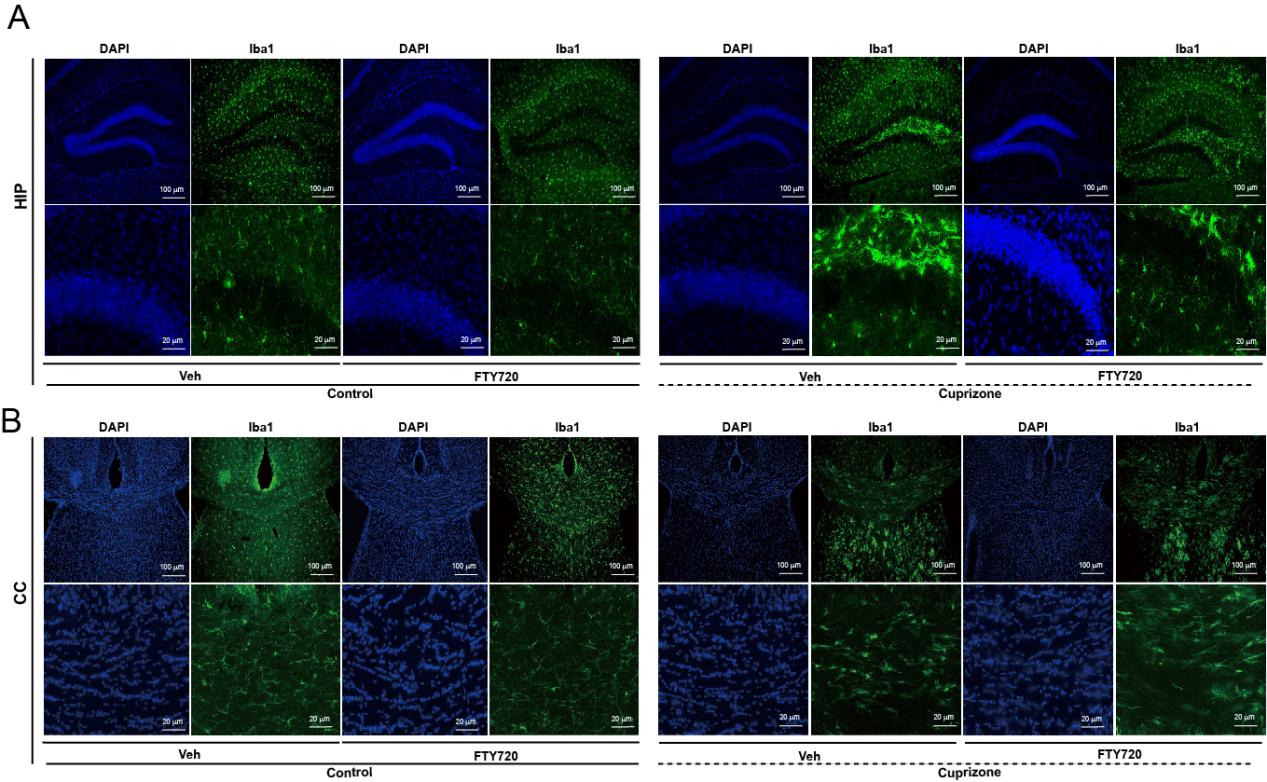


**Fig S3. Representative image of immunostaining in hippocampus and corpus callosum**

(A) Representative image of Iba1 immunostaining in the HIP. (B) Representative image of Iba1 immunostaining in the CC. Blue: DAPI nuclear stain, Green: Alexa488 (Iba1). An enlarged view of the top row is shown in the bottom row.


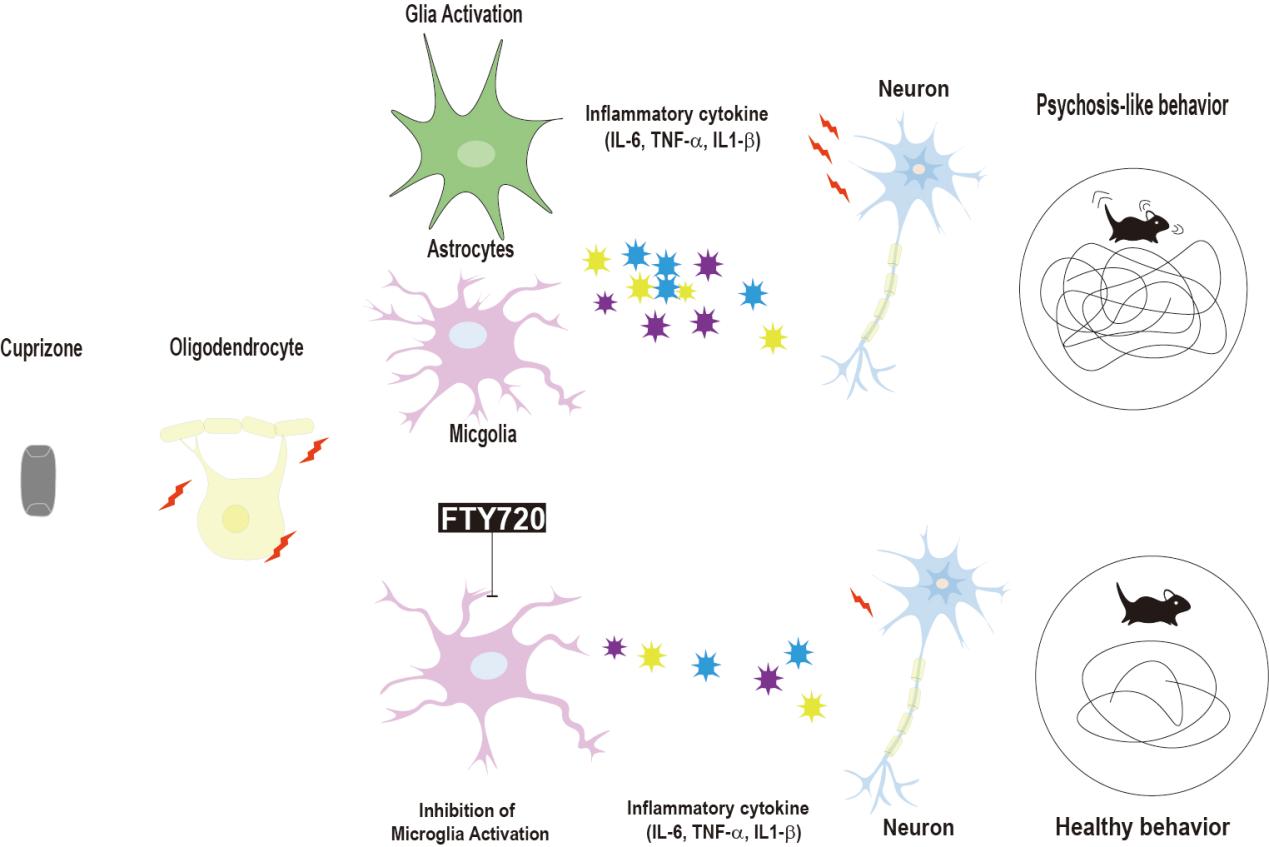


**Fig S4. Hypothesis of FTY720 mechanism of action in psychosis model mice.**

Short-term cuprizone exposure does not cause prominent demyelination, but specifically causes damage to oligodendrocytes. Such damage results in activation of glial populations including microglia, triggering the release of proinflammatory cytokines. Extracellular release of proinflammatory cytokines induces neuronal dysfunction and contributes to behavioral disorders such as psychosis. FTY720 administration inhibits microglial activation and regulates pro-inflammatory cytokine release, reduces neuronal damage, and improves psychosis-like behavior.
